# Effects of Black Soldier Fly (*Hermetia illucens* L., BSF) Larvae Addition on In Vitro Fermentation Parameters of Goat Diets

**DOI:** 10.3390/insects15050343

**Published:** 2024-05-10

**Authors:** Shengyong Lu, Shengchang Chen, Siwaporn Paengkoum, Nittaya Taethaisong, Weerada Meethip, Jariya Surakhunthod, Qingfeng Wang, Sorasak Thongpea, Pramote Paengkoum

**Affiliations:** 1 School of Animal Technology and Innovation, Institute of Agricultural Technology, Suranaree University of Technology, Nakhon Ratchasima 30000, Thailand; lsy05224810@gmail.com (S.L.); nittaya@gmail.com (N.T.); jariya@gmail.com (J.S.); sorasak@gmail.com (S.T.); 2 Institute of Animal Nutrition and Feed Science, Guizhou University, Guiyang 550025, China; 3 Program in Agriculture, Faculty of Science and Technology, Nakhon Ratchasima Rajabhat University, Nakhon Ratchasima 30000, Thailand; siwaporn.p@nrru.ac.th; 4 Institute of Animal Husbandry and Veterinary, Guizhou Academy of Agricultural Sciences, Guiyang 550005, China; qingfeng@gmail.com

**Keywords:** black soldier fly, methane, gas production, ammonia nitrogen, volatile fatty acids

## Abstract

**Simple Summary:**

The black soldier fly (BSF) contains protein, fat, chitin, and rich minerals and has the potential to be used as a protein feed source. The purpose of this experiment was to evaluate the effects of different levels of BSF on rumen in vitro fermentation gas production, methane (CH_4_) production, ammonia nitrogen (NH_3_-N), and volatile fatty acids (VFAs). The results have shown that the supplementation of 10% BSF yielded in vitro fermentation indicators similar to those of the non-supplementation group. Although supplementing BSF of 15% reduced methane production, it also reduced the concentrations of rumen VFA. Therefore, it is recommended to supplement the diet with 10% BSF instead of soybean meal.

**Abstract:**

The purpose of this experiment was to evaluate the effects of different levels of BSF on rumen in vitro fermentation gas production, methane (CH_4_) production, ammonia nitrogen (NH_3_-N), and volatile fatty acids (VFAs). The experiment comprised four treatments, each with five replicates. The control group contained no BSF (BSF0), and the treatment groups contained 5% (BSF5), 10% (BSF10), and 15% (BSF15) BSF, respectively. Results showed that at 3 h, 9 h, and 24 h, gas production in BSF5 and BSF10 was significantly higher than in BSF0 and BSF15 (*p* < 0.05). Gas production in BSF5 and BSF10 was higher than in BSF0, while gas production in BSF15 was lower than in BSF0. At 6 h and 12 h, CH_4_ emission in BSF15 was significantly lower than in the other three groups (*p* < 0.05). There were no differences in the pH of in vitro fermentation after BSF addition (*p* > 0.05). At 3 h, NH_3_-N levels in BSF10 and BSF15 were significantly higher than in BSF0 and BSF5 (*p* < 0.05). At 6 h, NH_3_-N levels in BSF5 and BSF10 were significantly higher than in BSF0 and BSF15 (*p* < 0.05). Acetic acid, propionic acid, butyric acid, and total VFAs in BSF0, BSF5, and BSF10 were significantly higher than in BSF15 (*p* < 0.05). In conclusion, gas production, CH_4_ emission, NH_3_-N, acetic acid, propionic acid, butyric acid, and VFAs were highest in BSF5 and BSF10 and lowest in BSF15.

## 1. Introduction

In recent years, with population growth and economic development, people’s demand for livestock products has been increasing [1,2], leading to higher and higher prices for protein feed ingredients [3]. Therefore, the production cost of animal husbandry is constantly increasing, and it is urgent to find new feed protein raw materials. Insects as protein feed are a current research hotspot. Currently, there are about 2000 species of edible insects, and the most widely used in animal feed are yellow mealworm (*Tenebrio molitor*) [4], cricket (*Gryllus bimaculatus*) [4], and BSF (*Hermetia illucens* L.) [5,6], because their edible portion is close to 100% [7]. BSF is rich in crude fat, protein, minerals, and amino acids [8]. Its average protein content can reach about 40% [9], which is equivalent to soybean meal [10], and can reach up to 65% [11], which is similar to the protein content of fish meal [12]. The crude fat content can reach 29–51% [7,13], and the saturated fatty acid lauric acid (C12:0) content can reach 40% [14,15]. Medium-chain fatty acids (C6:0-C12:0) are important in inhibiting methane production [16]. Many studies have shown that although C12:0 did not alter rumen VFA levels, it reduced rumen methane production, followed by a reduction in the number of methanogens and bacteria in the rumen [17,18]. In terms of minerals, calcium, phosphorus, copper, and potassium are the most abundant [7]; these minerals lower the pH and increase the molar ratio of rumen acetic acid, the ratio of acetic acid to propionic acid, and the concentration of total volatile fatty acids in the rumen fluid [19,20,21]. Interestingly, it is also rich in essential amino acids, with the highest levels of leucine, lysine, and valine, all higher than soybean meal and comparable to fish meal [22,23,24]. Based on the above nutrient content, BSF has potential as an alternative to animal protein feed.

The application of BSF in ruminants is still relatively small, and only a few pieces of literature have been written about the effect of BSF oil on rumen fermentation. Jayanegara et al. [25] showed that crickets, mealworms, and BSF could reduce CH_4_ production in vitro. Interestingly, BSF oil did not negatively affect rumen fermentation and also increased potentially health-promoting trans-11 18:1 without altering trans-10 18:1 concentrations [26]. BSF studies in dairy cows showed no negative effects on feed digestion. Still, they decreased rumen pH, increased VFA concentrations, improved yield, and altered milk fatty acids, most notably in the 10% supplementation group [27]. Previous studies have shown that BSF of different ages and the ratio of concentrated feed to roughage reduce the NH_3_-N and digestibility of dry matter organic matter (OM) in in vitro fermentation [28]. However, currently, neither ruminant in vivo nor in vitro studies have identified the optimal supplementation level of BSF. Therefore, the purpose of this study was to evaluate the effect of different levels of BSF on goat rumen fermentation in vitro and to provide a theoretical basis and data support for our next comprehensive research in vivo.

## 2. Materials and Methods

All goats were handled by the Rules of Animal Welfare of the Suranaree University of Technology (SUT; SUT 4/2558). The experiment was carried out at the SUT Goat Farm, Nakhon Ratchasima, Thailand (14°53′37.9″ N, 102°01′22.0″ E).

### 2.1. Rumen Fluid Collection

Four adult goats (17.74 ± 1.01 kg) were fed (dry matter basis) 50% corn silage, 15% corn, 15% cassava, 18% soybean meal, and 2% premix (DM 83.92%, crude protein 13.41%, ether extract 1.62%, NDF 42.50%, ADF 23.85%). The daily feed intake was 2.5% of body weight. The rumen contents of four goats were collected on the morning of the 7th day before feeding. A tube was inserted into the rumen from the mouth, and the rumen fluid was sucked out using a vacuum pump. The collected rumen contents were mixed and then filtered and separated with four layers of gauze. The obtained rumen fluid was transferred into a vacuum bottle and sent to the laboratory within 5 min as a culture medium.

### 2.2. Experimental Design and Fermentation Substrates

The experimental design was a single-factor, completely randomized design with 4 treatments and 5 replicates for each treatment. The control treatment did not contain BSF (BSF0), and the other treatments contained 5% (BSF5), 10% (BSF10), and 15% (BSF15) of BSF, respectively. The fermentation substrates were formulated according to NCR (2004), with a crude protein (CP) content of 14%. The experimental diet composition and chemical composition used in the treatment are shown in Table 1. The fatty acid composition of experimental diets is shown in Table 2.

### 2.3. In Vitro Fermentation

The in vitro cultivation medium’s composition was the same as that mentioned in Wei et al. [29]. In a nutshell, the following solutions were combined evenly: distilled water, artificial saliva, constant element solution, trace element solution, reducing agent solution, and resazurin solution. The ratios were 47.56%, 23.78%, 23.78%, 0.01%, 4.76%, and 0.11% based on volume. Each replicate (0.5 g) of the fermentation substrate was added to 50 mL of an inoculum consisting of the medium prepared above and rumen fluid in a 2:1 volume ratio, poured into a CO_2_ 125 mL vessel, snap-sealed with a deoxidizer, and placed in the incubator with a rubber stopper, a constant temperature of 39 °C, and a fluctuation frequency of 120 r/min. Gas collection tubes with calibration marks were used, and gas production was directly read and recorded at 3 h, 6 h, 9 h, 12 h, 24 h, 48 h, and 72 h. Fermentation products were collected at 3 h, 6 h, 9 h, and 12 h, terminated with ice, and pH was immediately checked with a pH meter (Mettler Five Easy Plus Series, Columbus, OH, USA). Two samples (1 mL) were collected; one was added with metaphosphoric acid (1% *w*/*v*; 1 mL) for VFA analysis, and the other was used for NH_3_-N analysis and stored at −20 °C. For methane analysis, 5 mL of headspace gas was collected from the fermentation flask using a gas-tight syringe (Hamilton, Reno, NV, USA).

### 2.4. Sample Analysis

#### 2.4.1. Chemical Composition of Feeds

The DM, ash, EE, and CP of the feed were determined according to the methods of the Society of Official Analytical Chemists [30]. NDF and ADF were determined by the method of Van et al. [31].

The content of minerals was detected according to Pieterse [32]. Briefly, add 5 mL of 6 mol L^−1^ hydrochloric acid to 0.5 g of the sample, put it in an oven at 50 °C for 30 min, take it out, add 35 mL of distilled water, then filter and make up to 50 mL. Minerals were measured on an iCAP 6000 series inductively coupled plasma (ICP) spectrophotometer (Thermo Electron Corporation, Strada Rivoltana, 20090 Rodana, Milan, Italy) equipped with a vertical quartz torch and a Cetac ASX-520 autosampler (Labtech, Beijing, China). TEVA Analyst software 1.6 was used to calculate mineral concentrations.

According to Tian et al. [33], the fatty acid composition was extracted using a chloroform–methanol solution. Using n-hexane as the internal standard, a gas chromatograph–mass spectrometer (GC-MS; Thermo Fisher Scientific, Waltham, MA, USA) was used as follows: injector temperature at 270 °C, detector temperature at 280 °C, and a programmed temperature starting at 100 °C for 13 min followed by a heating rate of 10 °C/min to reach 180 °C and maintained for 6 min. Subsequently, the temperature was increased at a rate of 1 °C/min to 200 °C and held for 20 min, followed by a final increase to 230 °C at a rate of 4 °C/min and maintained for 10.5 min. The carrier gas used was nitrogen with a shunt ratio of 100:1, and the sample volume injected was 1.0 μL. The assay conditions aimed to achieve a theoretical plate number (n) of at least 2000/m and a separation degree (R) of at least 1.25.

The amino acid content detection method was slightly modified according to Tian et al. [34]. Briefly, weigh 50 mg of the sample into a 20 mL hydrolysis tube, add 10 mL of 6 mol/L HCL, freeze it in liquid nitrogen, use a vacuum pump to pump to 7 Pa, and then fill with nitrogen for 1 min and tighten the tube cap. The hydrolysis tube was placed in a constant temperature drying oven at 110 °C for hydrolysis for 24 h, cooled, mixed, opened, and filtered. An appropriate amount of the filtrate was sucked with a pipette, placed in a rotary evaporator, and evaporated to dryness under vacuum at 60 °C, repeated twice. Add 5 mL of 0.2 mol/L sodium citrate buffer, shake well, and centrifuge at 12,000× *g* for 5 min at 4 °C (Allegra R X-30R Centrifuge, Beckman Coulter, Life Sciences Division Headquarters 5350 Lakeview Parkway S Drive Indianapolis, IN 46268, USA). The following were the UPLC conditions: individual AAs were separated using an ACQUITY UPLCR BEH C18 column (2.1 mm × 100 mm × 1.7µm, Waters, Milford, CT, USA) at 40 °C. The injection volume was 5 µL, and the mobile phases were A = 10% methanol (contains 0.1% formic acid) and B = 50% methanol (contains 0.1% formic acid). The gradient elution conditions were as follows: 0~6.5 min, 10~30% B; 6.5~7 min, 30~100% B; 7~8 min, 100% B. The following MS conditions were used: electrospray ionization source, positive ion ionization mode; ion power temperature of 500 °C, ion source voltage of 5500 V; collision gas pressure of 6 psi, curtain gas pressure of 30 psi; nebulization gas pressure and aux gas pressure were both 50 psi; and multiple-reaction monitoring scan mode.

#### 2.4.2. Ruminal Fermentation Characteristics

The method of Lee et al. [35] was used for the analysis of CH_4_. In brief, CH_4_ was evaluated using a gas chromatograph (Shimadzu GC-2010, Kyoto, Japan) outfitted with a thermal conductivity detector and a HayeSep Q 80/100 column (Restek, Bellefonte, PA, USA) at 90 °C. The sampler and detector were kept at a temperature of 150 °C. At a flow rate of 30 mL/min, argon was used as the carrier gas. The CH concentration of the samples was determined using a standard mixture of CH and CO (RIGAS, Daejeon, Republic of Korea).

The technique of Taethaisong et al. [36] was used to detect VFAs. Gas chromatography (Agilent 6890 GC, Agilent Technologies, Santa Clara, CA, USA), silica capillary column (30 m × 250 µm × 0.25 µm) was used to quantify the concentration of VFAs in the filtrate. The temperature was initially set at 40 °C for 2 min before rising to 100 °C at a rate of 3.5 °C/min and then to 249.8 °C at a rate of 10 °C/min. The whole duration of the run was 30 min. The boiling chamber temperature was 250 °C, and the carrier gas (99.99%) was He. Before columnization, the pressure was 31.391 psi, the carrier gas flow was 3.0 mL/min, and the solvent delay time was 3 min. The technique of [37] was used to detect ammonia nitrogen (NH_3_-N).

#### 2.4.3. Chitin Analysis

The chitin content of the BSF meal was analyzed following the method outlined by Liu et al. [38] with minor modifications. In brief, an aliquot of the prepupae meal (90–100 mg) was enclosed in an ANKOM filter bag (ANKOM Technology, Macedon, NY, USA) shaped to fit a 15 mL screw cap centrifuge tube. This aliquot underwent demineralization for 30 min in 5 mL of 1 M HCl at 100 °C. The demineralization process was followed by five washing steps in ASTM Type I water, ensuring neutrality. Subsequently, a deproteinization step was carried out in 5 mL of 1 M NaOH at 80 °C for 24 h. Finally, the sample was washed five times in ASTM Type I water until neutrality was achieved. After drying at 105 °C in an air-forced oven for 2 h, the chitin content (*CT*, g/kg DM) was calculated using the following formula:

$$CT=1000\times \frac{Fw-\left(Bw\times C\right)}{Sw}$$

where *Fw* = weight after demineralization, deproteinization, and drying (g); *Bw* = weight of the modified ANKOM extraction bag (g); *C* = dimensionless factor taking into account the MEAN weight loss of extraction bags (0.999, n = 6) treated according to the same procedure used for the samples; and *Sw* = exact amount of sample processed (g).

### 2.5. Statistical Analysis

Data were analyzed by one-way ANOVA with SPSS statistical software (Version 27.0 for Windows; SPSS, Chicago, IL, USA). The statistically significant differences were determined by Duncan’s multiple-range tests. Data were presented as the MEAN and SEM. The significance level was indicated at *p* < 0.05.

## 3. Results

### 3.1. Nutrition Facts of BSF

The approximate composition of BSF is shown in Table 3. It could be seen from the table that the dry matter content of the black soldier flies was very high, reaching 973.3 g/kg; the protein content was 407.4 g/kg, which was slightly lower than that of soybean meal (USA, 475.0 g/kg; Argentina, 460.0 g/kg; Brazil, 488.0 g/kg; India, 466.0 g/kg; Asia, 474.0 g/kg; China, 463.0 g/kg) [39]. The fat content was 327.0 g/kg. The minerals detected in this experiment include Ca, P, Na, Cu, and Se, while the content of P was much higher than that of other minerals, reaching 898.4 mg/kg.

### 3.2. Amino Acid Content of BSF

The amino acid content of BSF is shown in Table 4. A total of 16 amino acids were detected in BSF, of which there were nine types of indispensable amino acids, and the most abundant ones were arginine, leucine, lysine, and valine. From the data in the table, the content of indispensable amino acids was relatively balanced.

### 3.3. Fatty Acid Content of BSF

The BSF fatty acid content is shown in Table 5. BSF was rich in saturated fatty acids, of which the content of C12:0 was the highest, reaching 41.9 g/100 g, followed by C14:0 and C16:0, which were 6.80 g/100 g and 5.17 g/100 g, respectively. The C18 series has the highest content of unsaturated fatty acids; the content of c9 C18:1 was 7.58 g/100 g, but the content of C18:2n-6 was much higher than that of C18:3n-3, which were 9.39 g/100 g and 0.82 g/100 g, respectively. The content of SFA was 56.10 g/100 g, the total unsaturated fatty acid content was 18.50 g/100 g, the ratio of unsaturated fatty acid and saturated fatty acid was 32.98%, and the n-3 PUFA/n-6 PUFA was 10.4%.

### 3.4. Effect of Different Levels of BSF on Gas Production

**Figure 1:**
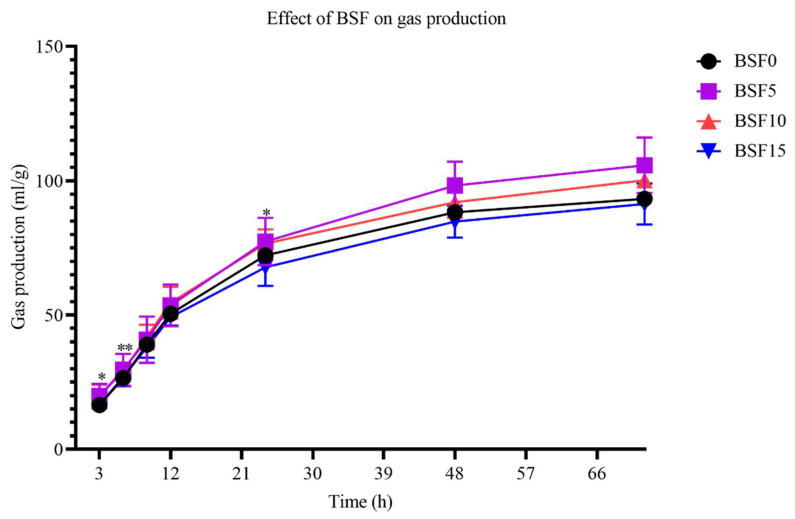
Effect of different levels of BSF on gas production. “*” means *p* < 0.05, indicating a significant difference; “**” means *p* < 0.01, indicating an extremely significant difference.

The influence of BSF on gas production is shown in Figure 1. From the 24th hour onwards, the gas production leveled off. At 3 h, 9 h, and 24 h, the gas production of BSF5 and BSF10 was significantly higher (*p* < 0.05) than that of BSF0 and BSF15. At 6 h, 12 h, 48 h, and 72 h, there were no significant differences (*p* > 0.05) in gas production among all groups. From the line chart, the gas production of BSF5 and BSF10 was higher than BSF0, and the gas production of BSF15 was lower than BSF0.

### 3.5. Effect of Different Levels of BSF on CH_4_ Production

**Figure 2:**
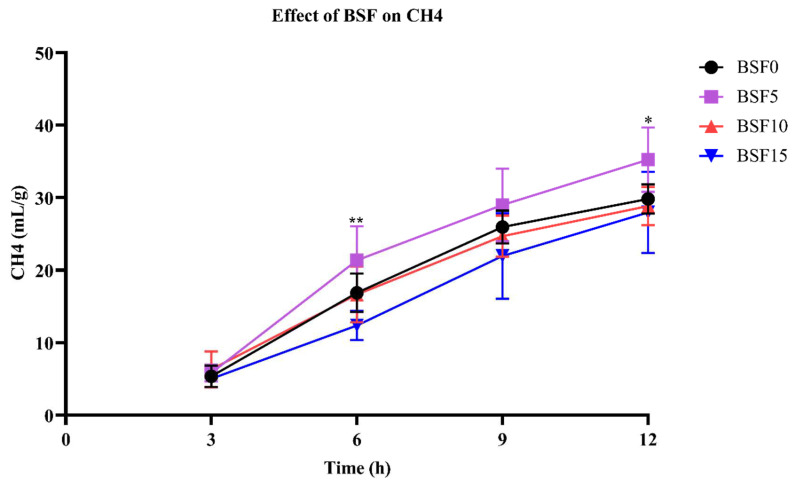
Effect of different levels of BSF on CH_4_ production. “*” means *p* < 0.05, indicating a significant difference; “**” means *p* < 0.01, indicating an extremely significant difference.

The effect of different levels of BSF on CH_4_ production is shown in Figure 2. From the 9th hour onwards, the CH_4_ production leveled off. At 6 h and 12 h, the CH_4_ of the BSF15 group was significantly lower (*p* < 0.05) than that of the other three groups. Overall analysis shows that the CH_4_ production of the BSF5 group was at the highest level, the BSF10 group was close to that of BSF0, while BSF15 was always at the lowest level.

### 3.6. Effect of Different Levels of BSF on pH and NH3-N

The effect of different levels of BSF on pH and NH3-N is shown in Table 6. There was no significant difference (*p* > 0.05) in in vitro rumen fermentation pH with BSF supplementation. At 3 h, the NH_3_-N levels of BSF10 and BSF15 were significantly higher (*p* < 0.05) than those of BSF0 and BSF5. At 6 h, the NH_3_-N levels of BSF5 and BSF10 were significantly higher (*p* < 0.05) than those of BSF0 and BSF15. At 9 h, the NH_3_-N level of BSF5 was significantly higher (*p* < 0.05) than that of BSF15. At 12 h, there was no significant difference (*p* > 0.05) in NH_3_-N levels among groups.

### 3.7. Effect of Different Levels of BSF on VFAs

The effect of different levels of BSF on VFAs is shown in Table 7. At 6 h, the acetic acid concentration of BSF15 was significantly lower (*p* < 0.05) than that of the other three groups, and there were no differences (*p* > 0.05) among the groups at 3 h, 9 h, and 12 h. At 3 h, BSF5 had the highest (*p* < 0.05) propionic acid concentration, there was no significant difference (*p* > 0.05) between BSF0 and BSF10, and BSF15 had the lowest (*p* < 0.05). At 6 h, the propionic acid concentration of BSF15 was significantly lower (*p* < 0.05) than that of the other three groups, and there were no significant differences (*p* > 0.05) in propionic acid concentrations among all groups at 9 h and 12 h. At 6 h, the butyric acid concentration of BSF5 was significantly higher (*p* < 0.05) than that of the other three groups, there was no significant difference (*p* > 0.05) between BSF0 and BSF10, and BSF15 had the lowest (*p* < 0.05) butyric acid concentration; during the other periods, there were no significant differences (*p* > 0.05) among groups. There was no significant difference (*p* > 0.05) in the A:P among the groups, but it had an increasing trend with BSF supplementation. In all periods, the total VFAs of BSF0, BSF5, and BSF10 were significantly higher (*p* < 0.05) than that of BSF15.

## 4. Discussion

We would like to know how BSF functions in the rumen of ruminant animals while considering the percentage of BSF supplementation. Additionally, recognizing that BSF supplementation may have detrimental effects under in vivo conditions, such as reducing VFA or NH_3_-N, we first conduct evaluations of in vitro fermentation.

### 4.1. Gas Production

In vitro gas production was originally used to predict ruminal degradability and metabolizable energy (ME) content of animal feed [40]; higher gas values indicate higher nutrient availability by rumen microbes [41]. This study showed that the gas production of the BSF5 and BSF10 groups was higher than that of the BSF0 group, and the gas production of the BSF15 group was lower than that of the BSF0 group. This shows that supplementing BSF at 5% and 10% was beneficial to improving the utilization rate of nutrients by microorganisms, while 15% had a negative effect on nutrient utilization, which may be related to the content of chitin and saturated fatty acids. Because the low level of chitin in the diet promotes the digestion of nutrients, as the number of additions increases, the digestibility of nutrients gradually decreases [42]. This theoretical study is confirmed by Jian et al. [43], who showed that supplementation levels above 15% with BSF reduced the apparent digestibility of nutrients. Tabata et al. [44] showed that the level of acid chitinase mRNA in bovine stomach tissue was very low. Belanche et al. [45] reported that adding different doses of chitin reduced the digestibility of organic matter. This is consistent with reports from Wencelova et al. [46], who found that chitosan reduced dry matter digestibility and total gas production. These results may be related to its antibacterial effect [47], whereby chitin interacts with negatively charged free fatty acids and thereby inhibits biohydrogenation in vitro, leading to changes in rumen protozoa populations [48]. In addition to chitin, SFA also contributes significantly to gas production. Both soybean oil and BSF have high individual saturated fatty acid content [49]. Soybean oil was characterized by high C16:0 content, while BSF was characterized by high C12:0 content, which will lead to reduced gas production. Jayanegara et al. [25] observed that C12:0 contained in the rumen has an inhibitory effect on gas production. Research studies on BSF reducing gas production were confirmed by Renna et al. [50].

### 4.2. CH_4_ Production

In this experiment, the methane production of the BSF15 group was the highest at 6 h and 12 h, and the CH_4_ production of the BSF0 and BSF5 groups was the lowest at 12 h, indicating that the digestibility of the diet of the BSF15 group was higher, which has the same result as the gas production. In this experiment, the CH_4_ production of the BSF5 group was slightly higher than that of the BSF0 group in the early stage, the BSF10 group was similar to the BSF0 group, and the CH_4_ production of the BSF15 group was always the lowest, which had the same result as the gas production. This is because high digestibility would lead to increased production of H_2_, which is the main substrate for methane formation by methanogenic archaea [51]. The reduced CH_4_ levels in the BSF15 group were related to the higher fat content, as numerous studies have shown the effect of fat supplementation on reducing CH_4_ emissions in ruminants [52,53]. In particular, medium-chain fatty acids (6–12 carbon atoms) had stronger inhibitory effects on methanogens and showed stronger CH_4_-mitigating effects, according to a meta-analysis by Yanza et al. [54] (41 studies) concluded that CH_4_ production per unit of digested organic matter decreased linearly under in vitro conditions, and tended to a quadratic decrease under in vivo conditions with increasing doses of MCFAs. Another reason for the reduction in the CH_4_ ratio may be related to chitosan, which is a biopolymer (N-acetyl-d-sugaramide) derived from the deacetylation of chitin. Belanche et al. [45] report that chitosan reduces methane emissions by 42%. In vitro, chitosan reduces methane emissions because of its antimicrobial properties, altering the structural bacterial community and shifting the mode of rumen fermentation to propionic acid production [55].

### 4.3. pH and NH_3_-N

In ruminants, rumen pH decreases a few hours after feeding and then increases again due to VFA removal, rumination, and salivation [56]. Rumen pH should include not only the average pH but also the pH range during the feeding cycle. In this experiment, supplementing BSF had no significant difference in the pH of in vitro fermentation. From the 3rd hour to the 12th hour, the pH fluctuated within a small range, demonstrating that BSF did not disrupt the internal balance of the rumen ecosystem. Microbial protein synthesis is mostly nitrogen-dependent, and ammonia can be the primary supply of nitrogen for bacterial development [57]. In the current experiment, at 3 h, the concentrations of NH_3_-N in the BSF10 and BSF15 groups were the highest. At 6 h and 9 h, the concentrations of NH_3_-N in the BSF5 and BSF10 groups were the highest. In general, the concentrations of NH_3_-N in the BSF5 and BSF10 groups were higher than those in the BSF0 and BSF15 groups. The high content of ruminal NH_3_-N in the high-concentrate diet is explained by a dynamic balance among NH_3_-N production and consumption by rumen bacteria [58,59]. The observation of large amounts of NH_3_-N in the stomach of ruminants indicates that the ruminants’ diet contains an adequate amount of accessible nitrogen, which was most likely due to the diet’s high energy concentration [57]. According to the research of Calabrò et al. [58], a higher rumen NH_3_-N concentration indicates a greater presence of rumen-degradable protein. Numerous studies have shown that added high levels of fat reduce ruminal NH_3_-N concentrations [60,61]. This may be related to a reduction in rumen protozoa since NH_3_-N was formed from bacterial protein degradation [62]. According to research by Castillejos et al. and Castillejos et al. [63,64], fats can interact with bacterial cell membranes, preventing the growth of specific strains and ultimately causing ammonia concentrations to drop. Another factor that may be important is that chitosan reduces NH_3_-N production by inhibiting protease activity and microbial deaminase [65]. According to a report from Kahraman et al. [66], NH_3_-N levels gradually decreased with BSF supplementation.

### 4.4. VFAs

VFAs are the major product of ruminal fermentation and are positively correlated with the digestibility of the substrate, accounting for approximately 40% to 70% of digestible energy intake [67]. The results of the current experiment show that, overall, BSF5 increased acetate, propionate, butyrate, and total VFAs; the BSF0 and BSF10 groups have similar results, while the BSF15 group decreased these indicators. On the one hand, it may be related to the mineral content in BSF because mineral supplementation increases the concentration of VFAs in the rumen [68]. Pino et al. [69] found that supplementation of minerals increased the concentration of VFA throughout the day in dairy cows. Wang et al. [70] reported that total VFA concentrations increased linearly and quadratically with increasing selenium supplementation. Trace minerals such as Zn, Mn, Cu, and Co are required for structural proteins, enzymes, coenzymes, and cellular proteins [71] and participate in many enzymatic processes in the rumen; they can alter microbial populations and metabolic pathways in the rumen, potentially leading to lower nutrient digestibility when micronutrient availability is limited [72]. However, high concentrations of minerals are less soluble in the rumen and bound less tightly to the ruminal solid digest [73], so the BSF15 group will reduce VFA production. On the other hand, the decrease in VFAs in the BSF15 group can be explained by chitin, crude protein, crude fat content, and fatty acid profile [74,75]. According to research from Ahmed et al. [76], who added 30% BSF to the diet, the total VFA output was lower than that of the group without supplementation. The results of the study by Jayanegara et al. [28] are similar. Supplementing 20% BSF resulted in lower VFA production than the SBM group. It may be because fatty acids can adsorb onto microbes in the rumen or feed particles, and their small molecules easily dissolve in the lipid layer of cell membranes, effectively causing physical damage to the cell membrane, disrupting energy metabolism and nutrient transport, leading to a decrease in the concentration of volatile fatty acids (VFAs) due to the death of cellulolytic bacteria [77]. Interestingly, probiotics and prebiotics can be used to increase VFA production [78]. BSF can grow well in harsh conditions such as food waste or animal waste, so BSF larvae contain a variety of microorganisms, such as lactic acid bacteria (LAB) [79]. Due to the special structure of chitin, many studies have shown its potential as a prebiotic [80,81]. However, based on the research on chitin in ruminants, it has the function of reducing the total VFAs [82], which may have a synergistic effect when digested together with other nutrients, which can become the focus of future research. In summary, supplementation with 15% BSF had a negative impact on ruminal VFA concentration.

## 5. Conclusions

Compared to the BSF0 group, the BSF5 and BSF10 groups increased gas production during in vitro fermentation, while the BSF15 group decreased it. CH_4_ production in the BSF5 and BSF10 groups was similar to that in the BSF0 group, while it decreased in the BSF15 group. Supplementation of BSF did not affect the pH of in vitro fermentation. Compared to the BSF0 group, the BSF5 and BSF10 groups increased the concentration of NH_3_-N, while the BSF15 group decreased the concentrations of rumen acetate, propionate, butyrate, and total VFAs.

## Figures and Tables

**Table 1:** Ingredients and chemical composition of experimental diets used in treatments.

Items	Treatments				SEM	*p*-Value
	BSF0	BSF5	BSF10	BSF15		
Ingredient, % DM						
Corn	60.0	60.0	60.0	60.0		
Soybean meal	25.0	20.0	15.1	10.5		
Rice bran	7.5	8.9	10.4	10.0		
Cassava	3.0	3.0	3.0	3.0		
BSF	-	5.0	10.0	15.0		
Soybean oil	3.0	1.6	-	-		
Limestone	0.2	0.2	0.2	0.2		
NaCl	0.3	0.3	0.3	0.3		
Premix ^1^	1.0	1.0	1.0	1.0		
Chemical composition, % DM						
DM	87.8	88.7	88.8	89.0	0.18	0.14
Ash	5.0 b	5.4 a	5.4 a	5.5 a	0.07	0.02
CP	13.3	13.9	14.2	14.0	0.16	<0.01
EE	4.0 b	4.5 ab	5.5 a	5.5 a	0.25	<0.01
NDF	44.9 a	41.8 b	42.5 b	43.5 ab	0.23	0.03
ADF	24.4	23.8	23.9	23.3	0.11	0.12

^1^ Contents per kilogram premix: 10,000,000 IU vitamin A; 70,000 IU vitamin E; 1,600,000 IU vitamin D; 50 g iron; 40 g zinc; 40 g manganese; 0.1 g cobalt; 10 g copper; 0.1 g selenium; 0.5 g iodine. ADF = acid detergent fiber, CP = crude protein, DM = dry matter, EE = ether extract, NDF = neutral detergent fiber. SEM = pooled standard error of treatment means. Different shoulder letters in the same row of data indicate significant differences (*p* < 0.05).

**Table 2:** Fatty acid composition of experimental diets used in treatments.

Fatty Acids	Treatments			
	BSF0	BSF5	BSF10	BSF15
C10:0	0.02	0.05	0.05	0.04
C12:0	0.00	2.20	4.19	6.29
C14:0	0.16	0.12	0.25	0.29
C16:0	14.67	14.18	13.96	12.68
C18:0	2.32	1.67	2.16	1.97
C18:1 c9	21.49	20.78	20.45	18.60
C18:2 n-6	40.47	37.84	37.07	32.06
C18:3 n-3	2.85	2.56	2.42	2.01
C20:0	0.10	0.04	0.05	0.03
C20:1	0.05	0.04	0.04	0.03
C22:0	0.06	0.04	0.04	0.02
SFA	17.33	18.30	20.70	21.32
UFA	64.86	61.29	59.97	52.69
n-6 PUFA	40.47	37.84	37.07	32.06
n-3 PUFA	2.85	2.56	2.42	2.01
n-3 PUFA/n-6 PUFA, %	7.04	6.77	6.53	6.27

PUFA = polyunsaturated fatty acid, SFA = saturated fatty acid, UFA = unsaturated fatty acid.

**Table 3:** The approximate composition of BSF.

Items	Contents
DM	973.3 g/kg
CP	407.4 g/kg
EE	327.0 g/kg
Ash	82.8 g/kg
CF	57.7 g/kg
Ca	33.9 mg/kg
P	898.4 mg/kg
Na	61.8 mg/kg
Cu	9.6 mg/kg
Se	0.5 mg/kg
Chitin	0.71 g/kg

CP = crude protein, DM = dry matter, EE = ether extract.

**Table 4:** Amino acid content of BSF (g/100 g).

Items	Contents
Indispensable amino acids	
Arginine	2.47
Histidine	1.43
Isoleucine	1.72
Leucine	2.88
Lysine	2.60
Methionine	1.27
Phenylalanine	1.69
Threonine	1.68
Valine	2.37
Dispensable amino acids	
Alanine	2.73
Aspartic acid	3.62
Cysteine	0.26
Glycine	2.33
Glutamic acid	5.31
Proline	1.87
Serine	1.65
Tyrosine	2.28

**Table 5:** The fatty acid content of BSF (g/100 g).

Items	Contents
C10:0	0.11
C12:0	41.9
C14:0	6.80
C15:0	0.04
C16:0	5.17
C16:1	0.49
C17:0	0.06
C18:0	0.99
c9 C18:1	7.58
C18:2n-6	9.39
C18:3n-3	0.82
C20:0	0.02
C20:1	0.05
C20:5n-3	0.16
C21:0	0.62
C23:0	0.39
SFA	56.10
MUFA	8.13
PUFA	10.37
TUFA	18.50
n-6 PUFA	9.39
n-3 PUFA	0.98
TUFA/SFA, %	32.98
n-3 PUFA/n-6 PUFA, %	10.4

PUFA = polyunsaturated fatty acid, SFA = saturated fatty acid, MUFA = monounsaturated fatty acid, TUFA = total unsaturated fatty acid.

**Table 6:** Effect of different levels of BSF on pH and NH3-N.

Items	Treatments					
	BSF0	BSF5	BSF10	BSF15	SEM	*p*-Value
pH						
3 h	6.96	6.99	7.14	7.13	0.15	0.13
6 h	6.78	6.66	6.59	6.62	0.25	0.71
9 h	6.94	6.93	7.00	7.01	0.12	0.63
12 h	7.00	6.95	7.04	7.04	0.10	0.56
Mean	6.92	6.88	6.94	6.95	0.11	0.83
NH3-N, mg/dL						
3 h	0.89 b	0.85 b	1.11 a	1.20 a	0.18	<0.01
6 h	1.13 b	1.51 a	1.34 a	1.01 b	0.22	<0.01
9 h	1.51 ab	1.64 a	1.43 ab	1.26 b	0.20	0.01
12 h	1.19	1.32	1.20	1.16	0.17	0.45
Mean	1.18 b	1.33 a	1.28 ab	1.16 b	0.10	0.01

SEM = pooled standard error of treatment means. Different shoulder letters in the same row of data indicate significant differences (*p* < 0.05).

**Table 7:** Effect of different levels of BSF on VFAs.

Items	Treatments					
	BSF0	BSF5	BSF10	BSF15	SEM	*p*-Value
Acetic acid (mmol/L)						
3 h	21.27	24.71	24.19	18.28	4.54	0.10
6 h	53.00 a	60.03 a	52.71 a	40.50 b	8.62	<0.01
9 h	67.96	68.18	69.15	62.22	5.41	0.19
12 h	72.78	72.97	74.35	66.23	5.82	0.13
Mean	53.76 a	56.47 a	55.10 a	46.81 b	4.88	<0.01
Propionic acid (mmol/L)						
3 h	7.44 b	10.00 a	8.01 b	6.00 c	1.73	<0.01
6 h	15.68 a	17.02 a	15.81 a	12.46 b	2.22	<0.01
9 h	18.15 a	20.47 a	18.45 a	15.58 b	2.31	<0.01
12 h	19.16 ab	20.04 a	19.25 ab	16.56 b	2.69	<0.01
Mean	15.11 b	17.38 a	15.38 b	12.65 c	2.65	<0.01
Butyric acid (mmol/L)						
3 h	7.75	7.91	7.65	7.26	0.49	0.21
6 h	9.85 b	10.46 a	9.18 bc	8.78 c	0.85	<0.01
9 h	9.23	9.84	9.24	8.85	0.79	0.26
12 h	15.43	16.02	15.06	14.82	0.80	0.11
Mean	10.57 ab	11.06 a	10.28 b	9.93 b	0.57	<0.01
A:P						
3 h	2.93	2.46	3.06	3.03	0.53	0.26
6 h	3.39	3.56	3.37	4.00	0.43	0.78
9 h	3.78	3.36	3.74	4.11	0.45	0.15
12 h	3.80	3.48	3.75	4.11	0.46	0.21
Mean	3.47	3.22	3.48	3.60	0.30	0.22
Total VFAs (mmol/L)						
3 h	36.47 ab	42.62 a	39.85 a	31.55 b	6.25	0.02
6 h	78.54 a	87.51 a	77.69 a	61.73 b	11.00	<0.01
9 h	95.35 a	98.49 a	96.84 a	88.65 b	7.14	0.03
12 h	107.37 a	111.04 a	108.66 a	97.61 b	7.56	0.02
Mean	79.43 a	85.92 a	80.76 a	69.38 b	6.65	<0.01

A:P = acetic acid:propionic acid, SEM = pooled standard error of treatment means. Different shoulder letters in the same row of data indicate significant differences (*p* < 0.05).

## Data Availability

All the other material is from published literature found in the References section.
